# The Effectiveness of Online Learning in Improving the Electrocardiogram Interpretation Skills of Junior Medical Trainees: A Mixed Methods Observational Study

**DOI:** 10.7759/cureus.42320

**Published:** 2023-07-23

**Authors:** Amr Elkammash, Mian W Ahmed, Mustafa Alsinan, Khaled Madi

**Affiliations:** 1 Cardiology, Cambridge University Hospitals NHS Foundation Trust, Cambridge, GBR; 2 Respiratory Medicine, Southampton General Hospital NHS Foundation Trust, Southampton, GBR; 3 Internal Medicine, Princess of Wales Hospital, Bridgend, GBR; 4 Cardiology, University Hospitals Dorset NHS Foundation Trust, Bournemouth, GBR

**Keywords:** lectures, covid-19, ecg, pandemic, teaching, skills, face-to-face, virtual, problem-solving, medical education

## Abstract

Introduction

The COVID-19 pandemic hindered medical education and limited access to clinical skills training for trainee medical doctors, including electrocardiogram (ECG) interpretation. These restrictions prompted a shift towards virtual training environments and online learning. In this study, we assessed the impact of the pandemic on trainees' confidence and their perceived difficulty in independently interpreting ECGs. Additionally, we examined the effectiveness of two online learning approaches, namely lectures and case-solving webinars, in improving their skills.

Methods

The study was a mixed methods observational study conducted in three phases. In the first phase, a cross-sectional study was conducted to subjectively assess the trainees' confidence levels and the perceived difficulty independently reading ECGs. The second phase involved a cohort study where an online learning module consisting of eight lecture-based sessions was implemented. This module covered all the topics recommended in the foundation doctor training curriculum. The third phase also involved a cohort study where an online case-based discussion learning module with two problem-solving webinars was introduced. We assessed the outcomes on a 1 to 10 Likert scale for confidence and perceived difficulty in independently reading ECGs.

Results

Sixty-five trainees participated in the initial cross-sectional study. Among them, 100% of the participants reported substantial difficulty in interpreting ECGs (scoring 6 or more on the Likert scale), and 76.5% of the participants did not feel enough confidence to read ECGs independently (scoring 6 or less). Ten trainees attended the second phase. Online lectures significantly increased the mean confidence score by 1.9 points (t(9) = 2.82, p = 0.02, 95% confidence interval (CI) [0.38-3.42]) and significantly reduced the mean of the perceived difficulty score by 2.7 points (t(9) = 5.71, p < 0.001, 95% CI [1.63-3.77]). Compared to the online lectures, the online problem-solving sessions significantly increased the mean of the composite score of confidence and perceived difficulty in reading ECGs (-0.8 vs. 4 points, 95% CI [1.49, 8.26], p = 0.011).

Conclusion

The COVID-19 pandemic negatively affected the ECG reading skills of junior medical trainees. However, the online teaching approach effectively improved their confidence and the level of difficulty they experienced in ECG interpretation. Applying online case problem-solving was found to be superior to the lecture-based approach in enhancing these parameters.

## Introduction

The COVID-19 pandemic had a negative impact on medical practice from different perspectives, such as the cancelation of appointments and elective procedures and limiting access to medical care [1]. Medical education also suffered from the pandemic, where the application of social distancing rules led to the cancelation of educational events and bedside teaching for medical students. These limitations drove a shift to online teaching platforms and virtual simulations [2]. In Cardiology, access to training materials became limited during the pandemic, even to Cardiology trainees and fellows [3]. Consequently, the junior medical trainees were expected to struggle more to access basic Cardiology training during the pandemic, such as training on reading electrocardiograms (ECGs), to use such skills in providing proper medical care.

In this study, we assessed the effect of the COVID-19 pandemic on the ECG reading skills of junior medical doctors (foundation doctors and senior house officers (SHOs)) in a secondary medical care facility (a district general hospital) in the United Kingdom (The West Suffolk Hospital NHS foundation trust). In addition, we also investigated the effectiveness of online teaching in providing ECG interpretation training and compared the different approaches of online education: lectures and case-based discussions.

## Materials and methods

Study design

**Figure 1:**
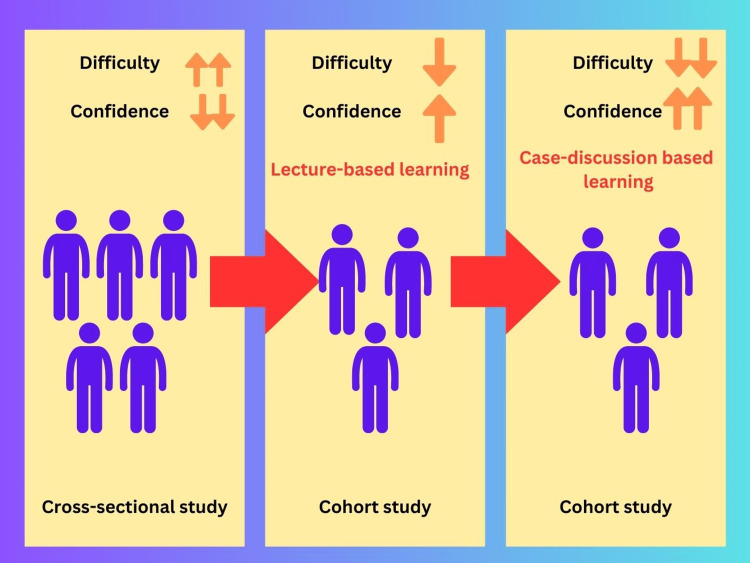
The central diagram of the study.

Our study was an observational pre-post intervention mixed methods study consisting of three parts. The first part involved an initial cross-sectional study to assess the baseline confidence levels and the difficulty experienced by trainees in reading ECGs. In the second phase, we conducted a cohort study where we implemented a lecture-based e-learning module and examined the changes in the study variables. Lastly, the third phase was also a cohort study, where we introduced a case-based learning module and evaluated the changes in the composite score of the ECG reading confidence scale and the trainees' subjective difficulty reading ECGs, comparing it to the lecture-based approach (Figure 1).

The study was conducted in a single district general hospital (a secondary healthcare facility that provides medical training services to graduate doctors at different levels of their careers) in the United Kingdom. We ran the study following the second wave of the COVID-19 pandemic from January 14 to October 30, 2022. The initial phase of the study (cross-sectional study) ran from January 14 to April 20, 2022; the second phase ran from April 21 to August 30, 2022; and the third phase ran from August 31 to October 30, 2022.

We used a 1 to 10 Likert scale to measure the level of confidence (a score of 1 represented the least level of confidence, and a score of 10 was the highest) and the level of difficulty the trainees experienced (a score of 1 represented the least perceived difficulty, and a score of 10 represented the highest) in the three phases. The questionnaire was circulated among the trainees using the Google Forms online application (Google LLC, California, United States) via the hospital email.

In the second phase, we implemented a virtual training model consisting of eight online lectures, each lasting one to two hours. These lectures provided the theoretical foundation for the various cardiovascular presentations mentioned in Table 1. We tested the participants' ECG reading skills with an online exam covering the topics that were taught in the lectures. During the third phase, we introduced a case-based learning module, conducting two case-based discussion sessions, each also lasting one to two hours. These sessions presented the same topics in the form of case studies within a clinical context. We used the Microsoft Teams (Microsoft Corporation, Redmond, Washington, United States) application to apply the modules. The study was reported according to the STROBE guidelines for reporting observational studies [4].

**Table 1:** The topics covered in the ECG e-learning course. IHD: ischemic heart disease, STEMI: ST-elevation myocardial infarction, NSTEMI: non-ST-elevation myocardial infarction, COPD: chronic obstructive pulmonary disease.

The topics covered in the ECG e-learning course
Normal ECG.
ECG in IHD: STEMI. NSTEMI. Old myocardial infarction. Stable angina. High take-off vs. STEMI Aortic dissection.
Bradyarrhythmias and heart block: Sick sinus syndrome. First-degree heart block. Second-degree heart block. Third-degree heart block. Junctional rhythm. Bundle branch and fascicular blocks.
Tachyarrhythmias: Atrial, junctional, ventricular.
ECG in systemic disorders: Pulmonary embolism. COPD/pulmonary hypertension. Systemic hypertension. Infiltrative cardiomyopathy/constrictive pericarditis. Renal failure. Subarachnoid haemorrhage. High intracranial pressure.
Congenital arrhythmogenic syndromes: Wolf Parkinson white. Arrhythmogenic right ventricular dysplasia. Brugada syndrome. Long QT syndrome. Short QT syndrome. Hypertrophic cardiomyopathy.
Other topics: Pacing cardiac resynchronization therapy. Pericardial effusion valvular heart disease: Aortic stenosis, mitral stenosis, infective endocarditis.

Participants

In our initial cross-sectional study, we included 65 junior medical doctors who were trained in the hospital (70% were foundation doctors, and 30% were SHOs). They were invited via the hospital electronic mail. In the second phase, we tested the lecture-based e-learning module on ten randomly selected junior doctors representing the different training stages (10% foundation year 1, 60% foundation year 2, and 30% SHOs). In the third phase, two participants dropped off, and the study was conducted on eight participants (25% foundation year 1, 25% foundation year 2, and 50% SHOs).

Statistical methods

We expressed the continuous variables as mean ± standard deviation (SD). We used the Spearman correlation analysis to assess the correlation between the different variables. We used the two-tailed student t-test to examine the hypothesis and compare the outcomes before and after the intervention. We used IBM SPSS Statistics for Windows, Version 29 (Released 2022; IBM Corp., Armonk, New York, United States) to run the statistical analysis. We considered a p-value of 0.05 or less statistically significant.

Power calculation

The post hoc power calculation of the cross-sectional study with the assumptions of alpha error of 5% and prevalence of reduced confidence in reading ECGs in junior medical trainees of 50% (as reported by McAloon et al. [5]) showed a study power of 99.7%. The post hoc study power calculation of the cohort study that assessed the benefits of the lecture-based e-learning module with the assumptions of alpha error of 5% and effectiveness of e-learning methods and reported mean endpoint Likert score of 7.25 (as reported by Patel et al. [6]) was more than 99.9%. The post hoc study power of the cohort study assessing the case-based approach was more than 99.9%, with an alpha error of 5% and a mean endpoint Likert score of 8.88, as reported by a later study [6].

Ethics statement

All the study procedures were compliant with the Helsinki Declaration of research ethics in human subjects. The local quality improvement and clinical audit committee approved the study and registered the study under the number 4324. We obtained participation consent from all the subjects included in the study.

## Results

**Figure 2:**
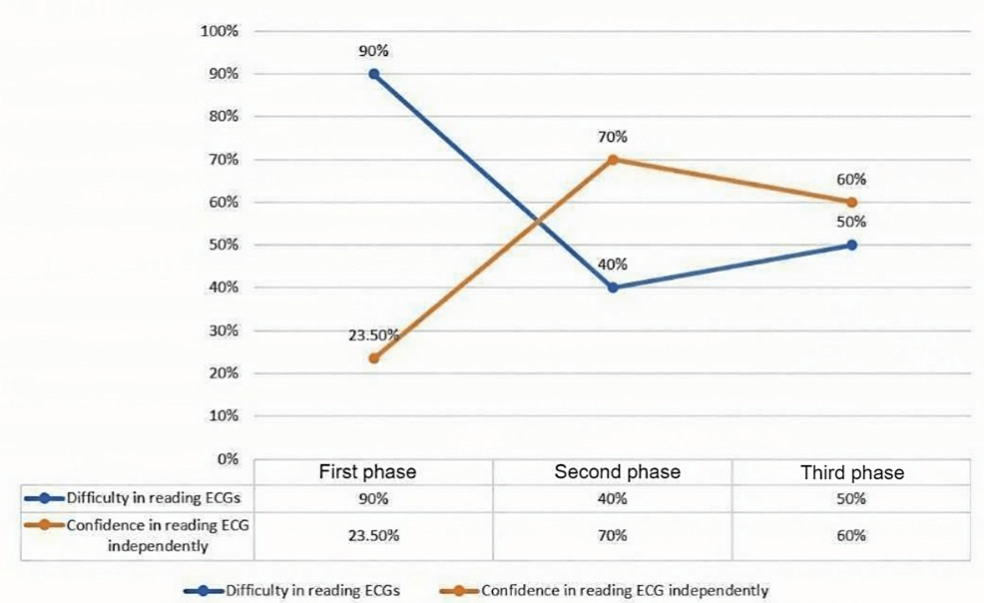
A summary of the study results across the different phases of the study.

The results of the study are summarized in Figure 2. 

The results of the initial cross-sectional study

Sixty-five trainees participated in the initial cross-sectional study. Of them, 100% of the participants felt substantial difficulty in interpreting ECGs (scoring 6 or more on the Likert scale), and 76.5% of the participants did not feel enough confidence to read ECGs independently (scoring 6 or less), with 34.3% lacking that confidence (scoring 4 or less). Furthermore, 72.3% of the participants preferred the online teaching method, mainly due to the flexibility of the method.

The result of the cohort study using the lecture-based teaching approach

**Figure 3:**
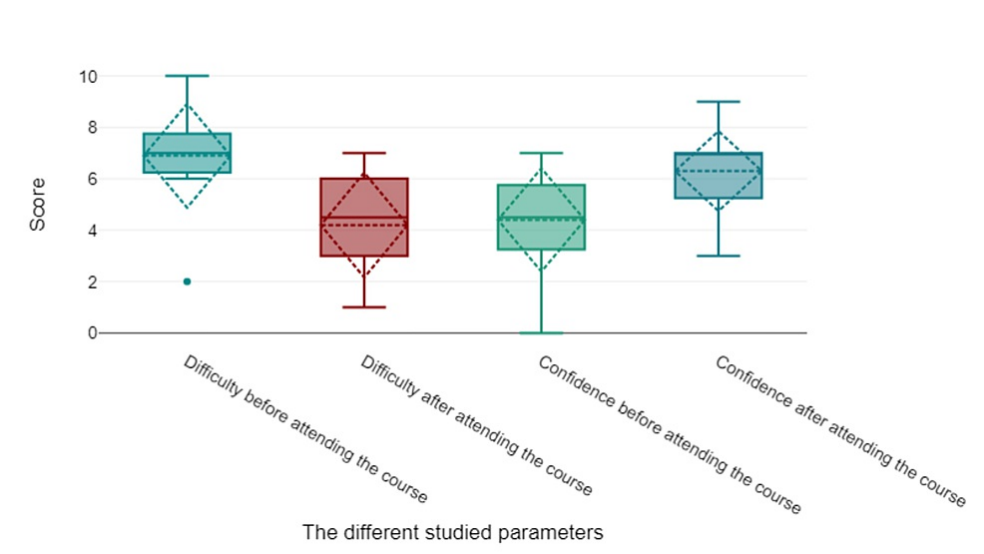
The effect of online lecture-based learning on the trainees’ confidence and perceived difficulty in reading ECGs.

The participants' confidence in the independent reading of the ECGs significantly improved after using the lecture-based online teaching model. The increase in the mean confidence score was 1.9 points (t(9) = 2.82, p = 0.02, 95% confidence interval (CI) 0.38-3.42) (Table 2). The perceived difficulty in reading ECGs has significantly decreased after attendance at the course. The decrease in the mean difficulty score was 2.7 points (t(9) = 5.71, p < 0.001, 95% CI 1.63-3.77) (Table 2 and Figure 3). The Spearman correlation analysis showed a high negative correlation between the perceived difficulty and the number of lectures attended by the participant (r = -0.63, p = 0.049). The participant's scores in the final ECG exam showed a high positive correlation with the number of lectures attended (r = 0.68, p = 0.031) rather than the trainee training stage (r = -0.29, p = 0.418).

**Table 2:** The confidence and the perceived difficulty scores among trainees at baseline and after attendance of the online lecture-based learning module.

Parameter	Before the course attendance	After the lecture-based module	p
The score of the self-confidence scale (mean±SD)	4.4 ± 2.12	6.3 ± 1.64	0.02
The score of the perceived difficulty scale (mean±SD)	6.9 ± 2.13	4.2 ± 2.15	<0.001

The result of the cohort study using the case discussion-based teaching approach

**Figure 4:**
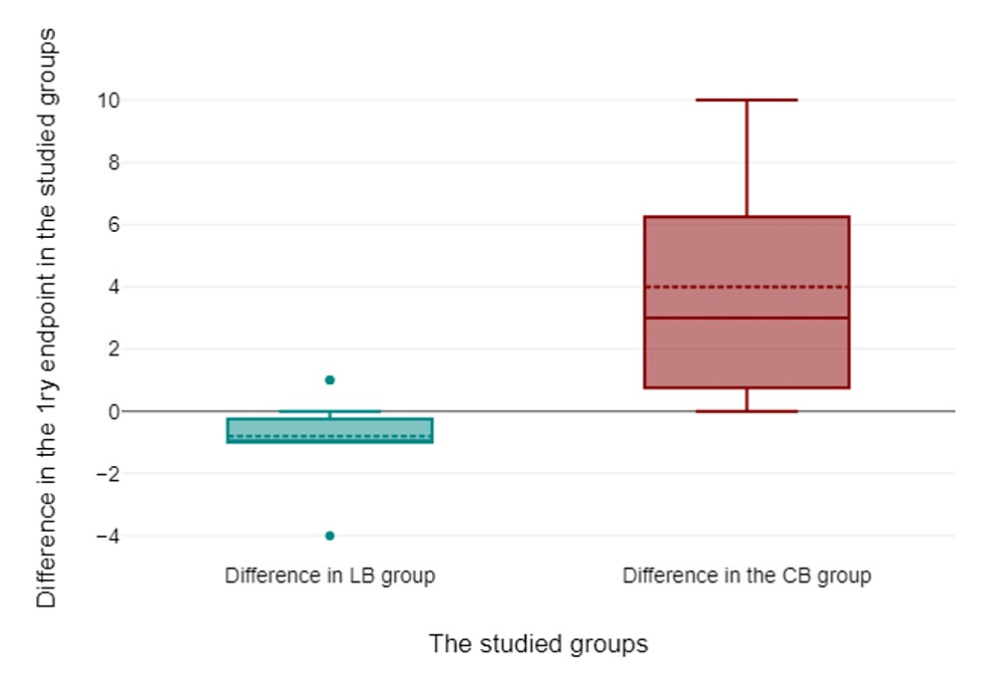
The composite score of self-confidence and perceived difficulty among trainees after the lecture-based and the case-based learning modules. LB: lecture-based learning; CB: case-discussion-based learning; 1ry: primary.

The mean of the composite score of self-confidence and perceived difficulty in reading ECGs was significantly higher after the case-based learning module than after the lecture-based module (4 points vs. -0.8 points, respectively, 95% CI [1.49, 8.26], p = 0.011) (Table 3 and Figure 4).

**Table 3:** The composite score of self-confidence and perceived difficulty among trainees after the lecture-based and the case-based learning modules.

Parameter	Lecture-based group (n = 10)	Case-based group (n = 8)
1: Composite score before the intervention (mean ± SD)	11.3 ± 1.34	7.75 ± 1.91
2: Composite score after the intervention (mean ± SD)	10.5 ± 1.35	11.75 ± 2.76

## Discussion

Our study showed the effects of the COVID-19 pandemic on the ECG reading and interpretation skills of junior medical trainees. It also demonstrated the effectiveness of online virtual training in building up such skills. We proposed that problem-solving case-based discussions were more effective in improving the trainees' confidence in reading ECGs independently. We showed that the magnitude of improvement in the trainees' skills was correlated with the number of attended sessions rather than the seniority.

Junior trainee doctors represent an educationally vulnerable group with difficulties in interpreting ECGs to provide proper healthcare [7]. Those difficulties extend even to junior cardiology fellows [8]. The COVID-19 pandemic made these training problems more apparent. The pandemic prevented the trainees from fulfilling their training requirements with prolongation of the time needed to complete them, affected their career progression, and blurred their career choices [9]. Our study suggested that the pandemic impacted the junior trainees' ability to deal with ECGs and interpret them confidently and independently. Such effects could be due to the lack of educational opportunities and events such as conferences, lectures, and journal clubs [9].

The study provided a solution to overcome the hurdles of ECG training for junior medical doctors: online learning in a virtual environment. This outcome converged with the previous results of Pourmand et al. [10], which revealed the usefulness of online learning in improving the ECG reading skills of emergency medicine trainees. Compared to face-to-face teaching, online ECG learning was non-inferior [11] or even superior [12] to it if the learning needs of the students were met. Uniquely, our study tested two different online learning approaches: conventional lecturing and case-based applied discussions. We found that the case-based discussions were superior to lectures in improving the ECG reading skills of junior doctors. To our knowledge, we were the first to test the different online learning models in ECG training. We found, contrary to the results of De Jager et al. [7], the ECG interpretation skills correlated directly with the number of training sessions attended rather than the seniority. Taken together, our results and the results of the previous studies point towards the ability of online case-discussion-based training to leverage the junior trainees' ability to read ECG efficiently.

Study limitations

Our study had a few limitations. We ran the study in a single hospital on a small number of trainees, which limits the generalizability of the results. However, our results matched with the previous broader scale studies. Another limitation was not running a baseline ECG test to establish the background trainees' abilities. This did not allow us to stratify the trainees' according to their background information to illustrate better the benefits gained after attending the course. A third limitation was the study's observational nature, with the non-randomization of the attendees and non-blinding of the investigators. Lastly, it could be useful if we stratified the trainees' based on their seniority to assess the effects of online education in the different stages of medical training.

## Conclusions

In conclusion, our study confirmed the negative impact of the COVID pandemic on junior doctors' ECG training. The study showed the usefulness of online learning in improving their ECG reading skills. The results were crucial in generating a hypothesis on the superiority of the problem-solving learning model over the lecture-based model in online ECG training. Wider-scale randomized studies are required to test this hypothesis further and provide solutions to improve medical training.
